# Development of a Modularized Seating System to Actively Manage Interface Pressure

**DOI:** 10.3390/s140814235

**Published:** 2014-08-05

**Authors:** Chung-Huang Yu, Tung-Yu Chou, Cheng-Huan Chen, Poyin Chen, Fu-Cheng Wang

**Affiliations:** 1 Department of Physical Therapy & Assistive Technology, National Yang-Ming University, No.155, Sec.2, Linong Street, Taipei 11221, Taiwan; E-Mails: b9221106@stmail.cgu.edu.tw (T.-Y.C.); scci58911120@hotmail.com (C.-H.C.); azxd32@hotmail.com (P.C.); 2 Department of Mechanical Engineering, National Taiwan University, No. 1, Sec. 4, Roosevelt Road, Taipei 10617, Taiwan; E-Mail: fcw@ntu.edu.tw

**Keywords:** pressure management, pressure ulcer, posture support devices, active support surface, cushion, seat simulator, modular design

## Abstract

Pressure ulcers can be a fatal complication. Many immobile wheelchair users face this threat. Current passive and active cushions do reduce the incidence of pressure ulcers and they have different merits. We proposed an active approach to combine their advantages which is based on the concept that the interface pressure can be changed with different supporting shapes. The purpose of this paper is to verify the proposed approach. With practical applications in mind, we have developed a modular system whose support surface is composed by height-adjustable support elements. Each four-element module was self-contained and composed of force sensors, position sensors, linear actuators, signal conditioners, driving circuits, and signal processors. The modules could be chained and assembled together easily to form different-sized support surfaces. Each support element took up a 3 cm × 3 cm supporting area. The displacement resolution was less than 0.1 mm and the force sensor error was less than 1% in the 2000 g range. Each support element of the system could provide 49 N pushing force (408 mmHg over the 3 cm × 3 cm area) at a speed of 2.36 mm/s. Several verification tests were performed to assess the whole system's feasibility. Further improvements and clinical applications were discussed. In conclusion, this modularized system is capable of actively managing interface pressure in real time.

## 1. Introduction

It is well known that preventing pressure ulcers is important and costs much less than treating them [1]. Pressure ulcer treatment is a long-term and expensive process, which often becomes a big burden to the patients and their family [2]. Even worse, pressure ulcers may cause death if proper daily care is not received. However, the occurrence of pressure ulcers around the world is still very high [3,4] even with precautions [5–7]. Consequently, there is a need to further study ulcer prevention.

The European Pressure Ulcer Advisory Panel (EPUAP) & National Pressure Ulcer Advisory Panel (NPUAP) provide recommendations for ulcer prevention in several aspects, including risk assessment, skin assessment, nutrition, repositioning, support surface, and so on [8]. Among them, many practical techniques are based on the notion that the primary cause of pressure ulcers occur would be ischemia by prolonged-high pressure on soft tissues [9,10] or damage to the microvascular system by friction and shearing forces generated by contact of bones or other solid materials [9,11].

Kosiak *et al.* [12] demonstrated that there was a negative correlation between the magnitude and duration of pressure on soft tissue for ulceration. In other words, if the pressure is over a threshold, ulcers would develop in a short period of time under high pressure and could be delayed under low pressure. In addition, different body areas have different pressure thresholds. Prominent bony body parts have low thresholds and are often the places where ulcers occur [13]. Furthermore, the risk areas are different for different postures. For example, both coccyx and ischial tuberosity are the most likely areas for pressure ulcers to occur while seated.

Through a self-protection mechanism, people would voluntarily or subconsciously change their posture or shift weight to bring the pressure down under the threshold. However, for those with breakage of this mechanism, such as reduction of mobility (e.g., spinal cord injured patients) or sensation (e.g., diabetics), external methods for reducing the magnitude and/or duration of pressure are needed to reduce the risk of ulcer development [14]. Apart from regular manual repositioning, advanced cushions for pressure management [15–17] are commonly applied.

Generally, cushions could be characterized as passive constant slow pressure (CLP) devices or active devices varying the pressure [17]. CLP cushions usually can be deformed or deflected to the shape of the users to distribute their weight over a larger area to provide low interface pressure, and they could be pre-contoured/custom-contoured and with different designs (e.g., spring-loaded support hardware) and constructions (foam, foam and air, foam and gel, viscous fluid and foam, air suspension, water suspension, *etc.*) [15,17]. There is no doubt that advanced passive skin protection cushions (SPC) [5] can lower pressure ulcer incidences and that “optimized” surface shape [6] can reduce peak interface pressure. However, one support surface shape cannot be always optimal due to (re)positioning or users' physical activities.

Active seating systems, which are suggested by EPUAP and NPUAP [8] for high-risk individuals unable or difficult to be repositioned manually, can redistribute and/or relieve pressure by changing their position/orientation or supporting shape. Commonly seen are alternating pressure seat supports and recline or tilt wheelchairs [17]. Alternating pressure devices are usually implemented by alternate inflation and deflation of air-fill cells. Their stability is questionable and it is not easy to customize their support surface shape for individuals. In addition, blindly alternating pressures not only can cause disturbance to the user, but also may cause unbearably high pressure on vulnerable or already damaged areas. As to recline or tilt wheelchairs, they may interrupt their user's activities.

**Figure 1.**
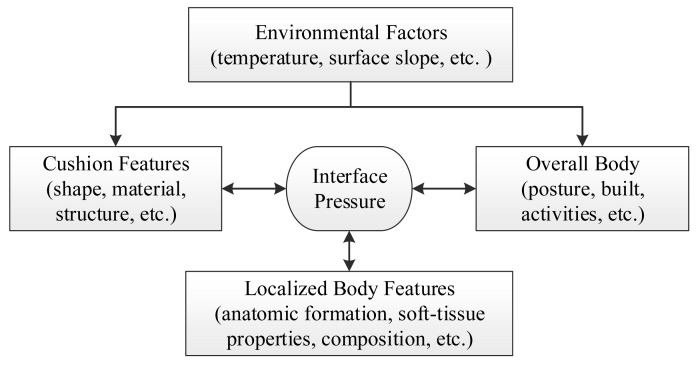
The factors interactively influence interface pressure distribution.

The role of a seat cushion can be considered from the aspects of the overall influencing factors and its underline biomechanics. From the outset, many factors can influence interface pressure distribution and they can grouped as environment, whole body, local body part, and seat features (see Figure 1).

The environmental factors may change the seat features in various aspects. For example, the internal pressure of air cushions will change with the temperature and hence the interface pressure. The whole body in different postures [18] or physical activities (e.g., raising arms, forward bending, *etc.*) can cause different force reactions from the support surface. Besides, the properties of the localized body part contacting with a support interface, such as anatomic formation, soft-tissue composition, and levels of near-by muscle contraction, *etc.* will interact with the support surface. Surely, the shape, material's properties (e.g., stiffness and resilience), structure, *etc.* of the cushion are the strong factors as well. All these factors interact each other in a complex and dynamic way.

From the viewpoint of biomechanics, the user's weight, his or her physical activities, and the reaction forces from the support surface(s) should keep equilibrium; and thus the soft tissues between the support surface(s) and skeletal structures will be under compressive and/or shear loading. Because of the anatomic formation and the connectivity of soft tissues and bones, the interface forces on the bones and those on the support surfaces are most likely to be different. Hence, while the interface pressures between the cushion and soft tissues are low, the internal interface pressures between bone and soft tissue could be high enough to cause deep pressure ulcers without superficial skin damage [19,20]. In addition, because of the complexity of soft tissue composition and variations of their mechanical properties, exact loadings on the every soft tissue from superficial to internal (subcutaneous) cannot be determined. Furthermore, not only large stress on soft tissues can be mechanically damaging, but also severe deformations can cause occlusion of the micro circular system [20].

In the light of these discussions, it can be seen that current CLP and active devices have addressed pressure management differently and partially. A system can keep constant low pressures in all situations and can actively redistribute and/or relieve them to further reduce the risk of pressure ulcer development would thus be very beneficial.

## 2. System Design and Implementation

### 2.1. Design Rationale

**Figure 2.**
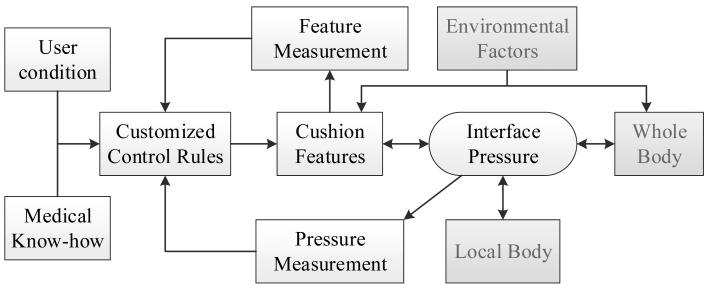
The system concept for real-time active pressure management.

As discussed above, interface pressure distributions result from many factors. Adjusting one factor could change the pressure distribution. Therefore it can be hypothesized that by adjusting a cushion feature properly in real time, good pressure management could be achieved even when the user is performing physical activities. Figure 2 shows the overall system concept. The interface pressures and a cushion feature are feedback to a real-time control system. Based on the feedback signals and control laws, the controller would adjust the cushion feature(s) to meet a desired pressure distribution. As a result, no matter in what postures and activities the user is, a good interface pressure can be maintained.

From the control engineering viewpoint, the influences from the other factors (gray boxed) can be treated as external disturbances to the system and can be compensated by feedback control. Among many cushion features, this study utilized the shape of support surface as the control parameter.

To change the shape of support surface, the design concept was as follows. A support surface, which is usually 2.5D, can be approximated by array of points. In other words, the system could be implemented by an array of retractable support elements. Noted that there can be only one point in the axial direction with this method and hence only 2.5D surfaces (not surfaces like spheres, donut shapes, *etc.*) can be formed.

In addition, for the sake of extendibility in seat size and ease of testing, debugging, and maintenance, modularization is an effective method. Hence, modular design was applied in the hardware and software.

### 2.2. Mechanical Design

As a potential posture support device, several practical issues needed to be considered. Firstly, the support surface for a seat may be under as high as about 200 mmHg pressure [21,22]. Hence, a system should sustain such loading and actively produce higher pressures/forces to change the shape under high loading. Furthermore, the pressure measurement should be less than 1% of the full loading range, *i.e.*, 2 mmHg. In addition to these requirements, it was wished that the spatial resolutions could be very high. Especially, the adjustment of height should be fine enough to cope with small deformations in bony areas.

**Figure 3.**
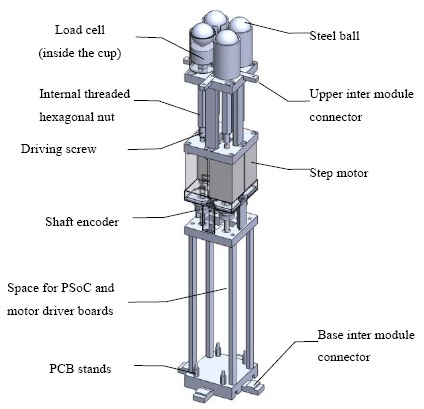
The mechanical design of a module unit in which threaded sleeves and driving rods form the retractable support elements.

After considering these design requirements and wishes against technology availability and feasibility, the system was implemented as follows: support elements were composed of M6 screws with 1 mm lead/pitch and hexagonal nuts (see Figure 3). By driving the screws, the nuts could move upwards and downwards and the heights of support elements could be changed. For ease of use and shape holding concern, 1.5 kg-cm stepper motors were utilized. The height of a support element could be calculated by using the number of steps and the thread lead. Because the lead was 1 mm and the stepper motor's resolution was 200 steps per revolution, the theoretical full step size was 0.5 μm. In addition, 10 count-per-revolution mechanical incremental shaft encoders (EVQ-WF100210B) were attached for each stepper motor to account for position errors such as missing steps and failure of position holding. After lead conversion, the shaft encoder used could in effect provide 0.1 mm measurement resolution of linear displacement. It should be noted that the 0.5 μm step size was much smaller than the 0.1 mm measurement resolution and could be used for open-loop positioning. To measure the force distribution under a user's buttock, each support element was equipped with FC2231 series load cells made by Measurement Specialties Inc. (Hampton, VA, USA) that were in the 10-pound range and with ±1% accuracy. On top of the load cell, a medium sized 1-inch diameter steel ball was placed to increase the buttock contact area and transfer the contact force to the small-tipped load cell.

### 2.3. Electronic System

**Figure 4.**
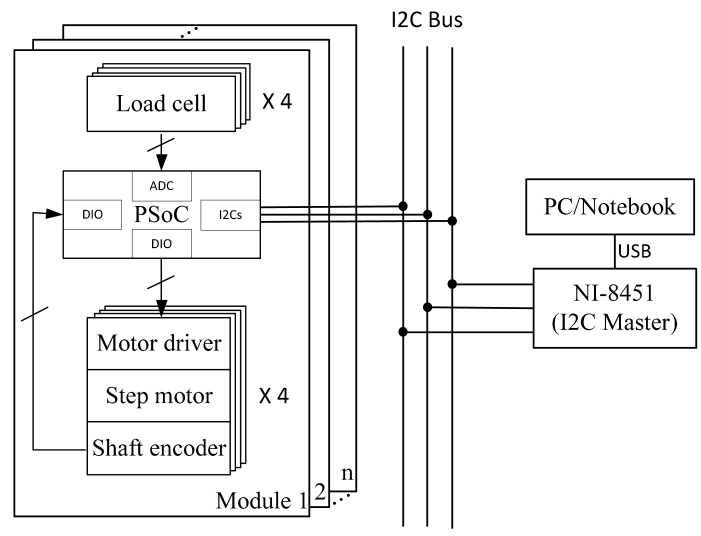
The structure of the electronic system.

The circuitry of the system is shown in Figure 4. Each module contained one PSoC1 chip from Cypress Semiconductor Corp. (San Jose, CA, USA) and four self-made stepper motor drivers built with A3983 DMOSE micro-stepping driver chips from Allegro MicroSystems, LLC (Worcester, MA, USA). The PSoC decoded the digital signals from the shaft encoders and converted the analogue signals from the load cells and output the driving signals to the motor drivers. As the FC2231 load cells have built-in Wheatstone bridges and amplifiers, the main PCB board for PSoC was quite compact. In addition, within the PSoC chip an I2C Slave was implemented to communicate with a PC. Since there was no built-in I2C interface in general purpose computers, the NI-8451 from National Instruments Corp. (Austin, TX, USA) was used as an I2C master for the computer to control the system.

### 2.4. Assembly Mechanism of the System

**Figure 5.**
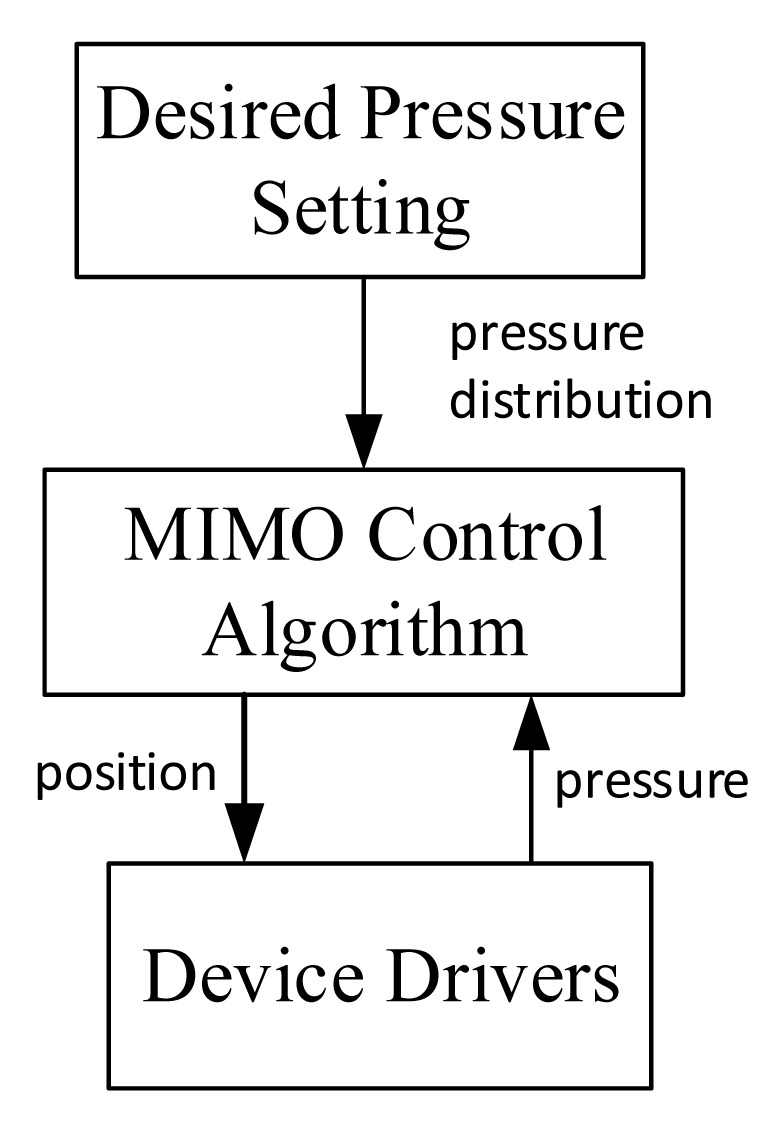
The proposed software architecture for pressure management.

For modular design, the system needed to be assembled easily. Mechanically, the modules could be connected with special designed connectors on grooves (see Figure 4). While assembling, upper connectors were screwed on adjacent modules and a base connector was tightly fastened between module bases. Electronically, the connection was achieved by “hooking” to the I2C bus (Figure 5).

### 2.5. Firmware and Software

The firmware on the PSoC chip was to bridge the PC and the local peripherals, *i.e.*, load cells, shaft encoders, and motor drivers. For the peripherals, the PSoC: (1) acquired load cell signals though the built-in ADC and converted them to force units; (2) decoded the shaft encoder and converted it to a displacement, and (3) commanded the motor driver through digital I/Os. For the PC, the PSoC prepared the sensor data for the PC to read and received commands from the PC with the I2C protocol.

The software on the PC was divided into three major levels (see Figure 5): desired parameter setting, control algorithm, and hardware driver subprogram. The desired parametera should be set according to users' characteristic, environment factors, and medical know-how. As to the control algorithm, it was required to accomplish multiple-input and multiple-output calculations according the desired pressure parameters and current sensor data. These two high levels of software require further clinical and engineering research, and were not fully implemented in this study. Nevertheless, several testing programs were written for system verifications as discussed in the following section. On the other hand, the low level part of the software was implemented with LabVIEW; the functions of the driver subprogram included to read individual sensor signals and to send motor driver command.

### 2.6. Support Element Characteristics

All the load cells used were tested with the following procedure: 50, 100, 200, 500, 1000 and 2000 g dead-weights were placed on top of the load cells, respectively, and this was repeated 15 times. The measured and the actual loadings were compared and the errors were less than 1% (20 g) over the 2000 g loading span.

**Figure 6.**
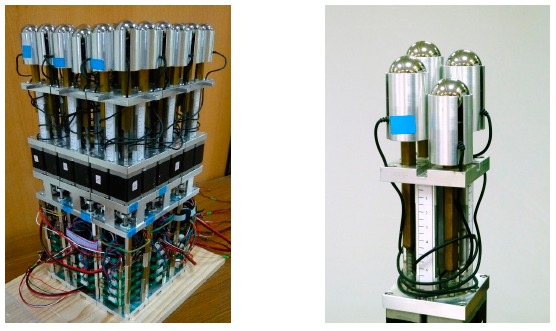
(**Left**) the active seating system composed of nine modules and a total of 36 support elements; (**Right**) A module unit with four support elements at different heights.

The system was designed to actively provide 49 N pushing forces for each support element. It was necessary to find the maximum pushing speed. Eleven different constant speeds (0.628, 0.90, 0.99, 1.08, 1.18, 1.31, 1.46, 1.975, 2.36, 2.94, 3.875 mm/s) were chosen for finding the maximum reliable speed. To begin with, a support element was randomly chosen to load with a 5 kg (near 49 N) dumbbell and underwent 3 cm up and 3 cm down displacements, respectively. To begin with the highest speed was used. If there was a missing step from the encoder reading, the next highest speed was set as the new highest speed and repeated the up-and-down test; if there was no missing step, the “current maximum speed” was set. The rest support elements went through with this procedure sequentially starting with current maximum speed. After all support elements finished the procedure, it was found that the maximum speed without missing steps for all support elements to extend 3 cm successfully was 2.36 mm/s. The developed modularized active support system (MASS) was shown in Figure 6 and the characteristics of the whole system are summarized in Table 1.

## 3. Experiments

To assess the feasibility of the MASS system, three kinds of tests were performed: “shape setting test”, “shape tracing test”, and “automatic force distribution test”.

### 3.1. Shape Setting Test

**Figure 7.**
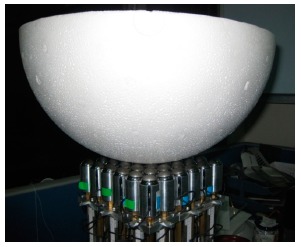
Setup for shape setting/tracing test.

The purpose of the shape setting test was twofold. One was to evaluate whether the overall system worked properly and the other was to simulate a support surface. The shape setting was straight forward by inputting the height of each support element. Two target shapes with spherical profiles of 15 cm and 19.8 cm radii were tested. There heights for every support element were calculated beforehand and input to the MASS. After the position resetting procedure, the MASS would set the desired displacement of each support element. After the MASS finished the setting, styrofoam half balls with specified radii (as in Figure 7) and 2 kg deadweight on top was placed to the simulated support surface. Then, the readings from the load cells could check whether the support elements were in contact with the specimens, *i.e.*, positive values meant for in contact. In addition, the readouts of all the load cells were summed up to check weight measurement errors. This procedure was performed three times for either specimen and the results shown in the Table 2 indicated that only one out of 216 (36 × 3 × 2) element-tests-shape was not in contact with the specimens and the measured weight was less than 1%. Note that the styrofoam balls were slightly deformable and an extra 2 kg deadweight was added in this test. If very stiff objects were used instead, more non-contact elements would be expected.

As the individual loadings on support element were measured, this could be used as a seat simulator to test the performance of a support surface shape.

### 3.2. Shape Tracing Test

**Figure 8.**
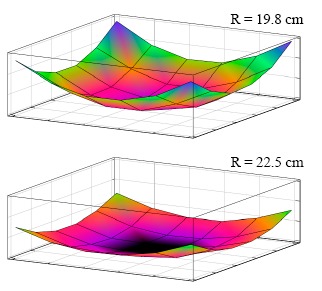
The traced profiles for styrofoam half balls with different radii.

The main purpose of this test was to evaluate whether the system could automatically trace the shape of an object. A simple program to trace out the shape of a specimen with a known shape and weight. The specimens were made by two styrofoam half balls with 19.8 cm and 22.5 cm radius, respectively, and with 2 kg dead weight on top of them (Figure 7). The specimen was put upon the initially flat shaped support surface and the support elements were sequentially elevated row-by-row and one-by-one. When the raising support element in contact with the specimen, *i.e.*, the load cell readout was positive, it stopped. After each support element went through this procedure, the program stopped and the summation of load cell readings were added together. Each specimen was tested three times, and it was found that the weight measurement errors were less than 1% and the shape did match the sphere shapes of the specimens (see Figure 8). However, it was found that the centers of specimens moved sideways after each test. This was because the support elements were moved sequentially rather than simultaneously in our simple test program.

### 3.3. Automatic Force Distribution Test

**Figure 9.**
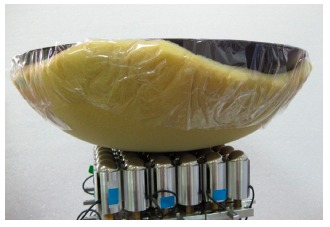
Setup for automatic pressure distribution test.

**Figure 10.**
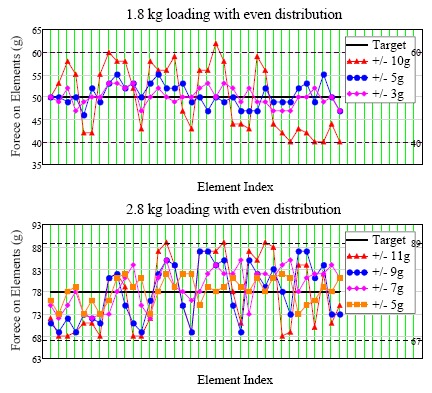
The results of automatic pressure distribution test with even distribution (the dash-lines indicate the upper and lower tolerance limits).

The “automatic force distribution test” was to evaluate the system's feasibility for active pressure management. Two kinds of pressure distributions were tried: even and arbitrary distributions. The arbitrary distributions were manually assigned and were symmetrical to the center of the seat and the support elements in the same module would target the same load for simplicity. The specimen for this test was made by a covered wok with 1 cm thin sponge underneath (see Figure 9). The purpose of the sponge was twofold. Firstly, it was used to estimate the effect of human's soft tissue around bony prominences. Secondly, it was used as the buffer between the two rigid bodies, *i.e.*, the steel balls and the wok, to make the load change less sensitive to the displacement of support element. Two different weights (1.8 kg and 2.8 kg) with different tolerance ranges were tested for both distributions. Again, a simple testing program was written. The program would continue adjusting the height of each support element till either all load cell readings came within the targeted load range or time limit was reached. Table 3 and Figure 10 show the results of the even distribution tests. For the loading of 1.8 kg, each of the 36 support elements should sustain 50 g; and for 2.8 kg, each support element should sustain 77.8 g.

It could be seen that as the tolerance decreased and the errors to target load were decreased; however, more iteration and more time were required to reach desired loading range for tighter tolerance. It was noted that one value (62 g) was outside the tolerance range. This could be because of signal noise. In addition, when the tolerance was too tight, the system would not settle down. This was because the load change was still too sensitive to the 0.1 mm position change (*i.e.*, 20 motor steps) of the support element. The solutions to this could be reducing the displacement change (*i.e.*, fewer steps) per iteration when fine tuning was necessary or inserting more soft material between the specimen and the system. The force mappings of the arbitral distribution settings are shown in Table 4 and Table 5 1.8 kg and 2.8 kg loading, respectively; the row-column position corresponded to the physical locations of each support element. Table 6 showed the iterations required to reach different specified loading tolerances.

**Figure 11.**
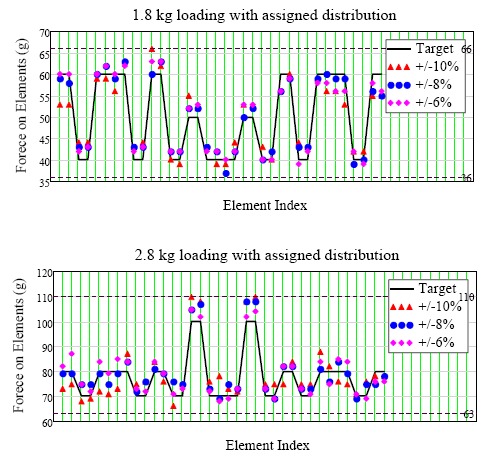
Results of automatic force distribution test with arbitral set distribution (the dash-lines indicate the upper and lower tolerance limits).

A tolerance below 4% could not be reached in this system because the sponge under the specimen was very thin and 0.1 mm step displacements (20 motor steps) were used, which could be further reduced by fewer motor steps. The system could automatically reach the targeted force distribution as shown in Figure 11. As the tolerance was reduced (marked as purple diamonds), the distributions were closer to the target values.

## 4. Discussion

### 4.1. System Features

From the mechanical point of view, a 0.1 mm step displacement of the support elements generally should be sufficient for the demands of a seating application, but for bony prominences, this may be not true since the soft tissues on these sites may be very thin and 0.1 mm displacement changes can produce a significant change of pressure variation as found in our “automatic pressure distribution test”. In addition, the support surface of the MASS was composed of steel balls, and the actual contact area with a human body may be very small and high pressure could occur. Therefore, the shape and material of the contact surface should be further modified for human application. As to the speed of shape change, 2.36 mm/s it should be quick enough to regulate the interface pressure to prevent pressure ulcers. On the other hand, high-speed shape changes are not desirable since they will compromise the user's seating stability.

### 4.2. System Improvement

Although they came from different application objectives, our MASS and the computer-aided seating system (CASS) developed by Brienza's team [23] are very similar in mechanical designs. Either system had its own features and they could be pointed out for future improvements.

#### 4.2.1. Pressure/Force Measurement

The pressure sensors on top of the swiveling heads of the support elements in the CASS can measure normal interface pressures, while our MASS measures the axial component of the interface forces. The pressure sensors in the CASS do not fully cover the top head and could underestimate the actual interface pressures. Although the MASS only measures the axial loading, the normal loadings could be estimated with additional data of the shape gradient/tangent of the support surface. However, its accuracy depends the magnitude of the shear/friction force. It is suggested that 3-axis load cells are instrumented to directly measure the 3 dimensional interface forces, since shear/friction forces have significant impacts on pressure ulcer development and direct measurement of the shear forces on the interface can bring to another important horizon of pressure ulcer studies.

#### 4.2.2. Shape Envelope

Both the MASS and the CASS can approximate 2.5D shapes rather than true 3D surfaces. Unlike our MASS which has about 40 mm high adjustment range, the CASS can provide 148 mm range which has the capability to form lateral and/or posterior support to increase seating stability. However, direct increases of the shape gradient should be avoided because large parallel relative motions between adjacent support elements can cause larger stretches and shear on the soft tissues in between. Changing the orientation arrangement of support elements, *i.e.*, lateral and posterior units toward to the buttock, can be a possible solution. The 30 mm (L) by 30 mm (W) by 0.1 mm (H) spatial resolution of our MASS is higher than that 40 mm by 40 mm by 1 mm of the CASS and can form a smoother and better fitting surface. As our test showed, with the 0.1 mm resolution in vertical displacement, the MASS can reach the desired loading within 6%, *i.e.*, ±2.4 g of 40 g which loading equivalent to averaged 0.2 mmHg over the 30 mm by 30 mm area. However, due to the spherical head of the support element, the actual pressure must be much higher, while the CASS with its flat swiveling head can provide more even pressure distribution which is a better design. Another concern is that due to the gaps between the supporting heads, the continuity of interface pressure distribution cannot be maintained and this has a bad influence on the localized superficial tissues. Extra cushions put on top of the support elements, either as a single piece or multiple pieces on individual heads, can reduce the effects of the gaps and the above-mentioned parallel motions between adjacent support elements. Overall higher density of support elements is suggested, especially the bony area, to better conform to the anatomic form.

#### 4.2.3. System Design

The later developed MASS took advantage of newly available technologies and design concepts. The modular design concept, either for hardware or on software, makes the system easier to debug, maintain, and extend. Each module is equipped with a “slave” microcontroller (PSoC). This enables all analogue/digital IO performed on site to increase the signals' integrity. In the future several functions could be implemented in the module, including high-speed IO, feedback control (e.g., PID control of targeted pressure/force), real-time measurement of soft tissue stiffness [24], material property simulation (e.g., spring), and so on. This distributed computing strategy frees a “master” computing platform to high level calculation of complex global optimization algorithm, e.g., [24]. The modular design of MASS is applied not only in software but also in hardware, makes the active support surface flexible in size and configuration.

### 4.3. Clinical Applications

Like the CASS, the MASS can be used as a research and computer-aided-manufacturing tool for cushion shape design [6,25]. These systems in their own right can be used as a posture support devices (PSDs) and be more effective for pressure management than conventional devices. However, as a PDS, all the functionalities including posture control, stability, comfort, and pressure management [16] should be considered collectively. Although the trade-off between these is beyond the scope of this study, the possible applications of an active system like the MASS and the CASS are worth further discussion.

Firstly, the pressure distribution can be automatically adjusted in real time to reflect the changes in environmental factors and users' physical activities. Pressure distribution can be converted into an optimization problem as shown in [6,25]. Although a detailed formulation of this problem will be discussed elsewhere, it is worth mentioning that at least the following points should be considered: (1) allowing less stiff tissues to sustain larger pressure [6,25], (2) if possible, enlarging the contact area to utilize more soft tissue to share the loadings, (3) setting upper pressure limits to prevent tissue damage in a short time, (4) setting upper pressure gradient limits because pressure gradients cause shear on soft tissue [26], noting that the boundaries between supporting and un-supporting areas should be considered as well, (5) setting cut-out areas (no pressure zones) where at high risk or already injured. Because of the high computing power available nowadays, the optimized pressure distribution could be calculated in a short time and hence the shape of support surface could accordingly be adjusted in real time.

The second application for pressure management by the MASS is to actively redistribute and/or relieve the pressure. This can be achieved, for instance, by adding time-varying pressure variations to the optimized pressure distribution. However, the magnitude of the addition should be big enough for pressure relief, but not too big to cause instability, and the frequency should be high enough before tissue damage but not too high before the underneath pressure being relieved and to cause disturbance to the user.

Previous studies [27,28] provide valuable information as a starting point to set parameters for these applications. For example, [29] showed that the pressures in healthy capillaries typically range from 15 to 25 mmHg, but would be lower for individuals with poor health. The loadings over this magnitude would cause the blood flow reduced or blocked and affect the perfusion. When overloaded for a long duration, soft tissues would be damaged due to inadequate perfusion. However, in practice the suggested pressures between support surface and the soft tissue range from 32 to 35 mmHg [30,31] which is higher than the capillary pressures. Hence, it is advised to perform pressure relief at least once every two hours for immobilized patients to reduce the risk of pressure sore development [31]. All these and others can be formulated as pressure management strategies. However, further detail studies are required to determine the best practice these applications by the MASS.

## 5. Conclusion and Future Work

In summary, this study has developed a modularized active support system and its active and automatic interface pressure management feasibility was verified. Several possible mechanical improvements are suggested for future development, including flat swiveling heads, increased density of the support element array, cushions on top of the support elements, *etc.* Among them, 3-axis load cell instrumentation in individual support elements is particularly important, because it can additionally provide information about shear interface forces and can have significant impact on pressure ulcer studies. F work would include the improvement and implementation of the hardware and software for practical clinical applications, performing subsequent clinical trials for validation.

## Figures and Tables

**Table 1.** System characteristics.

Force measuring range (Pressure over 3 cm × 3 cm area)	0 ∼ 43.8 N (365 mmHg)
Force measuring resolution (Pressure over 3 cm × 3 cm area)	0.0098 N (0.082 mmHg)
Forece sensor errors (Pressure over 3 cm × 3 cm area)	<20 g (1.6 mmHg)
Support area per element	30 mm × 30 mm
Full displacement of a support element	40.9 mm
Theoretical step size	0.5 μm
Displacment measurement resolution	0.1 mm
Rated speed (under 5 kg loading)	2.36 mm/s
Rated pushing force	49 N (408 mmHg)
Force and Posistion data update rate	> 360 scan/s/module unit
Module dimension	60 mm × 60 mm × 450 mm
Module weight	2.5 kg

**Table 2.** Shape setting test results.

**Specimen/Weight**	R15 cm/2280 g *			R19.5 cm/2200 g *		
		
Non-contact element (s)	0	0	1	0	0	0
Measured weight	2289	2293	2288	2211	2223	2233
Measurement errors	0.39%	0.57%	0.35%	0.5%	1%	1%

* The weights of the styrofoam half balls were 280 g and 220 g respectively.

**Table 3.** Results of automatic force distribution test with even distribution.

**Distribution**	**50 g Per Element (Total 1.8 kg)**			**77.8 g Per Element (Total 2.8 kg)**				
		
Tolerance (g)	±10	±5	±3	±11	±9	±7	±5	±3
Iterations	31	43	58	34	42	39	48	× *
Measured Weight	1791	1815	1804	2800	2797	2848	2898	× *
Weight error	−0.5%	+0.83%	+0.22%	0%	−0.1%	+1.7%	+3.5%	× *

* The symbol “×” means no solution was found within an iteration limition.

**Table 4.** Force map of arbitral distribution setting under 1.8 kg loading.

60	60	40	40	60	60

60	60	40	40	60	60

40	40	50	50	40	40
40	40	50	50	40	40

60	60	40	40	60	60
60	60	40	40	60	60

**Table 5.** Force map of arbitral distribution setting under 2.8 kg loading.

80	80	70	70	80	80
80	80	70	70	80	80

70	70	100	100	70	70
70	70	100	100	70	70

80	80	70	70	80	80
80	80	70	70	80	80

**Table 6.** Required iterations for automatic force distribution with arbitral load and different tolerance settings.

**Tolerance**	**10%**	**8%**	**6%**	**4%**
Iteration (1.8 kg)	9	11	15	× *
Measured Weight	1807	1820	1820	× *
Weigh Error	+0.38%	+1.1%	+1.1%	× *

Iteration (2.8 kg)	8	13	13	× *
Measured Weight	2860	2871	2864	× *
Weight Error	+2.1%	+2.5%	+2.2%	× *

* The symbol “×” means no solution was found within an iteration limition.
